# The effect of fenofibrate on early retinal nerve fiber layer loss in type 2 diabetic patients: a case-control study

**DOI:** 10.1186/s12886-018-0769-3

**Published:** 2018-04-18

**Authors:** Rui Shi, Lei Zhao, Yun Qi

**Affiliations:** 1 Department of Ophthalmology, Shaanxi Provincial People’s Hospital, No.256 Youyi west Road, Xi’an, 710068 Shaanxi Province China; 2 Department of Molecular Physiology and Biophysics, Holden Comprehensive Cancer Center, University of Iowa Carver College of Medicine, Iowa City, IA 52242 USA; 3 Department of Ophthalmology, the First Affiliated Hospital of Xi’an Jiaotong University, Xi’an, 710061 Shaanxi Province China

**Keywords:** Diabetic retinopathy, Fenofibrate, Retinal nerve fiber layer, Optical coherence tomography

## Abstract

**Background:**

Previous studies suggested that use of fenofibrate could significantly reduce the rate of progression into diabetic retinopathy (DR), and that retinal nerve fiber layer (RNFL) loss, which has been considered an important indicator for retinal neurodegeneration, might precede microvascular changes. The aim of this study was to assess the effect(s) of fenofibrate on RNFL thickness at early stage of DR in patients with type 2 diabetes mellitus (DM).

**Methods:**

In this retrospective matched case-control study we included a cohort of 89 patients with type 2 DM, aged 40 or above, between Jan 1, 2017 and March 31, 2017. Among the subjects, 48 patients received fenofibrate therapy and the other 41 patients did not receive fenofibrate treatment. We defined use of fenofibrate as the presence of any prescription for fenofibrate within 1 year before or any time after the diagnosis of DM, and all the patients had either no DR or non-proliferative diabetic retinopathy (NPDR). The fibrate users were well matched with non-fenofibrate users for gender, age and axial length. The RNFL thickness in all quadrants of both eyes was examined with spectral domain optical coherence tomography (SD-OCT). The multiple linear regression analysis was used to assess the association of RNFL thickness with potential risk factors of DR other than fenofibrate use.

**Results:**

The non-fenofibrate users had significantly reduced RNFL thickness of the superior quadrant of the right eye compared to the fenofibrate users (*t* = 2.384*, P =* 0.019). On the contrary, BMI (*p* = 0.034) and ACR (*p* = 0.024) were both negatively correlated to the RNFL thickness of the right eye.

**Conclusion:**

Oral administration of fenofibrate was suggestively associated with thicker RNFL in superior quadrant of the right eye of patents with early DR.

## Background

Diabetic retinopathy (DR) is the leading cause of blindness in patients with diabetes mellitus (DM) and considered as a microvascular retinal disease [1]. Previous studies have reported that increased serum cholesterol and triglyceride concentrations were associated with the development and severity of DR [2, 3]. Fibrates are peroxisome proliferator-activated receptor alpha (PPARα) agonists, which have been reported to effectively delay the progression of DR [4–6]. However, this benefit did not persist [7], and the potential mechanism of early DR remains unclear.

The most up-to-date studies have indicated that retinal neurodegeneration preceded microangiopathy of the retina and occurred at the earliest stage of DR [8–10]. Neuronal apoptosis and reduction in thickness of the inner retinal layers have been considered to cause defects in dark adaptation and contrast sensitivity, disturbances in color vision and abnormal microperimetry [11]. Topical administration of statins, the most widely used type of lipid-lowering reagents, has been proven to have a potent effect on preventing retinal neurodegeneration induced by DM [12]. The effect(s) of fibrates, another type of commonly used lipid-lowering drugs, on early retinal neurodegeneration still need further investigation.

The introduction of optical coherence tomography (OCT) has provided a useful tool for performing high-resolution imaging of the retina and subsequently measuring the thickness of the retinal nerve fiber layer (RNFL). The decreased RNFL thickness has been considered an important indicator for retinal neurodegeneration [13]. By using OCT several research groups have found that retinal thickness was decreased in diabetic patients without DR or with minimal DR compared to normal controls [14–17], and this decrease was associated with some risk factors, such as glycemic variability and vitamin D deficiency [13, 18]. However, the exact role(s) of these systemic risk factors in the development of retinal neurodegenerative lesions in DR remains largely unknown due to limited data. In the present study, we aimed to investigate the effect of fenofibrate on RNFL loss at early stage of DR and to explore the possible mechanism(s).

## Methods

### Participants and grouping

A total of 89 patients diagnosed with type 2 diabetes at age 40 or above were recruited from the Department of Endocrinology at Shaanxi provincial peoples’ hospital between Jan 1, 2017 and March 31, 2017, among whom were 48 fibrate users and 41 non-fibrate users. Use of fibrates was defined as regular or intermittent administration of fenofibrate for at least 1 year at any dosage. We also retrieved their medication history of taking sulfonylurea and insulin. We included patients who used fenofibrate before or after their diagnosis of diabetes; they either did not have DR or just had non-proliferative diabetic retinopathy (NPDR), but none of them had proliferative diabetic retinopathy. The fenofibrate users were well-matched with those who did not use fenofibrate for gender, age and axial length. We excluded patients younger than 40 years because they were unlikely to receive lipid-lowering reagents [19]. We also excluded patients who had one of the following situation: glaucoma, a positive family history of glaucoma, a refractive error of more than SE + 5 or SE − 3 diopters [13], AL > 25 mm in at least one eye [20], previous refractive surgeries, intraocular surgery, significant media opacity, a history of uveitis or retinal disease and neuro-ophthalmic disease. The patients who had received statins or any other type of lipid-lowering agents regularly were also excluded.

### Ophthalmological examinations

We identified all patients with careful examinations of both eyes by ophthalmologists. A detailed review of medical and ocular histories was also carried out for each patient. The fundus was examined with a handheld lens (90D Volk Optical) before the slit-lamp test. DR was graded blindly based on the Early Treatment Diabetic Retinopathy Study (ETDRS). Peripapillary RNFL (pRNFL) thickness was measured with 3D scan OCT imaging (6.0 × 6.0 mm, 512 × 128, 3D OCT-1, ver.8.30, Topcon Corporation, Tokyo, Japan) after pupillary dilation. Only the well-focused and well-centered images with quality strength of 25 or more and without eye movement were analyzed. The superior, inferior, nasal, temporal and average RNFL thickness was measured and then subjected to further analysis. The axial length was measured for three times with Zeiss IOLMaster 500 (Germany). The intraocular pressure (IOP) of all participants was measured with a non-contact tonometer (Tomey FT1000, Japan) for three times. Before each use, the tonometer was calibrated in accordance to the user’s manual and only the measurements with errors < 5% were used for data analysis.

### Laboratory tests

All included participants were diagnosed with type 2 DM according to the following criteria: a fasting plasma glucose level of 7.0 mmol/L or above or symptoms of diabetes plus a casual blood glucose level of 11.1 mmol/L or above [21]. Oral fenofibrate dosage and duration were collected from both medical records and questionnaires performed by ophthalmologists to ensure the accuracy of data. We defined regular fenofibrate use as a prescription obtained within 1 year after the date of diagnosis. Irregular use was any other use of fenofibrate for at least 1 year. The serum lipid profiles, including total cholesterol (TC), triglycerides (TG), high density lipoprotein cholesterol (HDL-C), low density lipoprotein cholesterol (LDL-C), glycated hemoglobin (HbA1c) and albumin/creatinine ratio (ACR) were collected from patients’ medical records.

### Statistical analysis

Statistical analyses were performed using GraphPad Prism 7 (GraphPad Software Inc., USA) and SPSS for Windows version 21.0 (SPSS, Inc., Chicago, IL, USA). Data were presented as mean ± standard deviation (SD) of each group. The independent-sample *t*-test was carried out to compare the means of two groups. The multiple linear regressions analysis was performed to identify factors potentially related to the RNFL loss; in this assay the RNFL thickness in superior quadrant of the right eye was the dependent variable, and the independent variables included age, gender, DR status, diabetes duration, serum lipids, ACR, AL, IOP and the duration of fenofibrate use. A *p* value < 0.05 was defined as statistically significant.

## Results

### Basic characteristics of the subjects

According to the inclusion and exclusion criteria, a total of 89 participants (178 eyes) with type 2 DM, aged 40 or above, were included in this study, among whom were 48 fibrate users and 41 non-fibrate users. At baseline, compared to the non-fibrate users, patients who had received fenofibrate treatment had a longer diabetic duration, higher BMI, increased blood TC and TG and lower HDL levels. No significant differences were discovered between the two groups in term of age, gender, AL, IOP, DR status, ACR and medication for DM treatment. The basic clinical and laboratory characteristics of all participants were summarized in **(**Table 1**)**.

**Table 1:** Basic characteristics of the participants

Characteristics	Non-fibrate user	Fibrate user	*t/x* ^*2*^	*p*
Subjects (N)	41	48	N/A	N/A
Age (yrs)	58.80 ± 12.68	57.71 ± 12.90	0.396	0.693
Women (%)	46.4	37.7	0.646	0.421
Diabetes duration (yrs)	10.39 ± 6.91	7.16 ± 4.92	2.514	*0.014
AL of the right eye (mm)	23.32 ± 0.40	23.45 ± 0.52	1.304	0.195
AL of the left eye (mm)	23.44 ± .0.56	23.61 ± 0.64	1.322	0.189
IOP of the right eye (mmHg)	19.11 ± 3.32	18.47 ± 4.01	0.811	0.419
IOP of the left eye (mmHg)	18.77 ± 3.99	18.34 ± 4.17	0.494	0.622
BMI(kg/m^2^)	24.63 ± 2.62	25.74 ± 2.43	−2.044	*0.044
Laboratory findings				
HbA1c (%)	8.52 ± 0.29	8.46 ± 0.28	0.991	0.324
Total cholesterol (mmol/l)	4.43 ± 0.21	4.75 ± 0.16	−8.148	*0.000
Triglyceride (mmol/l)	2.20 ± 0.28	3.02 ± 0.21	−15.76	*0.000
LDL (mmol/l)	2.74 ± 0.14	2.81 ± 0.12	−1.817	0.072
HDL (mmol/l)	1.27 ± 0.04	1.21 ± 0.05	6.177	*0.000
ACR (μg/mg creatinine)	23.15 ± 35.72	27.49 ± 44.64	−0.500	0.618
DR status (%)				
No DR	34.1	35.5	2.335	0.311
Mild NPDR	48.7	57.7		
Moderate NPDR	17	6.6		
Diabetes treatment (%)				
Sulfonylurea	62.5	58.3	0.035	0.852
Insulin	12.5	16.6	0.065	0.798

Each participant who used fenofibrate was exactly matched with a non-fibrates user for gender, age and DR status. *BMI* body mass index, *DR* diabetic retinopathy, *HbA1c* glycated hemoglobin, *HDL* high-density lipoprotein, *LDL* low-density lipoprotein, *RNFL* retinal nerve fiber layer, *AL* axial length, and *IOP* intraocular pressure. Data represented the mean ± standard deviation (SD) of each group. * *p* < 0.05. *p* < 0.05 was considered statistically significant. Diabetes duration, BMI, total cholesterol, triglyceride, HDL were found significant difference between groups

### The RNFL thickness

**Fig. 1:**
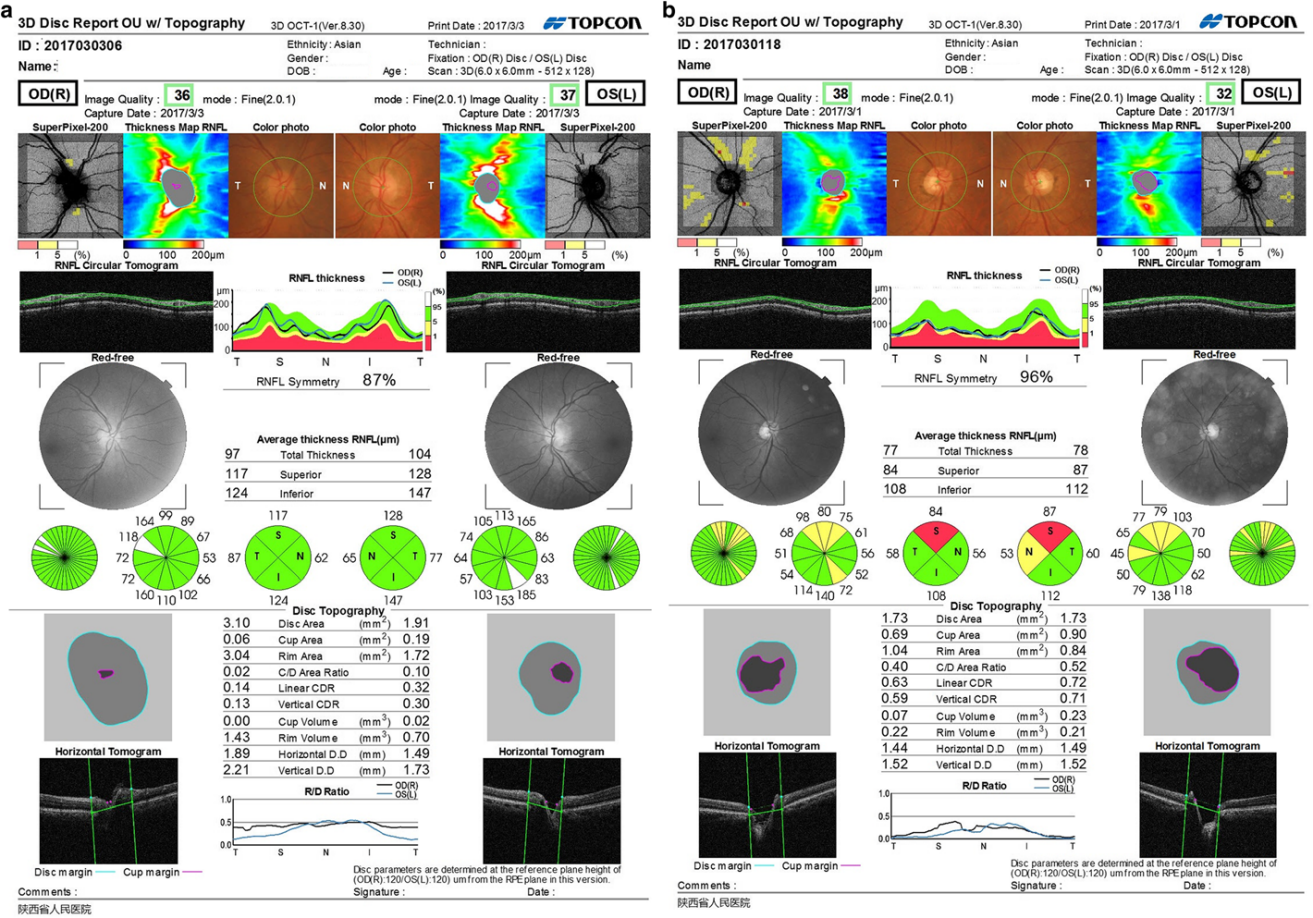
**a** and **b**. Representative images of the OCT test for both eyes in two matched patients. (**a**) The OCT image of a patient who use fenofibrate regularly. (**b**) The OCT image of a matched non-fenofibrate user. In both images, green, yellow and red colors represented within normal range, threshold thickness and occurrence of RNFL thinning, respectively

The RNFL thickness of the superior quadrant of the right retina of the non-fibrate users was significantly thinner (*t* = − 2.384, *P* = 0.019) than that of the fenofibrate users, which can be seen in the pictures of SD-OCT (Fig. 1a and b). However, no significant differences were discovered in the RNFL thickness of the superior quadrant of the left eye and in the average, inferior, temporal and nasal RNFL thickness of both eyes between the two groups (Table 2).

**Table 2:** The peripapillary RNFL thickness of fibrate user and non-fibrate user

Eyes	Non-fibrate users (*n* = 41)	Fibrate users (*n* = 48)	*t*	*p*
Right eye				
Superior quadrant	111.8 ± 3.596	123.0 ± 3.083	−2.384	*0.019
Inferior quadrant	120.4 ± 3.148	130.0 ± 3.925	−1.863	0.066
Nasal quadrant	70.95 ± 3.065	69.94 ± 2.252	0.271	0.787
Temporal quadrant	78.12 ± 3.021	76.02 ± 1.844	0.605	0.547
Average	95.89 ± 2.611	99.08 ± 2.192	0.940	0.351
Left eye				
Superior quadrant	116.7 ± 3.701	125.5 ± 3.262	−1.788	0.077
Inferior quadrant	124.7 ± 3.397	132.4 ± 3.985	−1.072	0.286
Nasal quadrant	72.51 ± 2.547	69.10 ± 2.127	1.035	0.303
Temporal quadrant	73.32 ± 1.59	72.38 ± 1.504	0.429	0.668
Average	96.95 ± 2.386	99.90 ± 2.089	−0.932	0.353

*RNFL* retinal nerve fiber layer. Data was presented as means ± SD of each group. *P < 0.05. *P* < 0.05 was considered statistically significant between the fibrate user and non-fibrate user group

### The potential risk factors for loss of RNFL thickness

To determine whether other risk factors, in particular blood lipids levels, were involved in the thinning of the right eye RNFL, a multiple linear regression analysis was employed and the results were shown in Table 3. Fenofibrate use was positively associated with the RNFL thickness of the superior quadrant of the right eye (*p* = 0.042). On the contrary, BMI (*p* = 0.034) and ACR (*p* = 0.024) were both found negatively correlated with the RNFL thickness of the right eye. However, no relationship between blood lipids levels and RNFL thickness was established in the analysis.

**Table 3:** Association between fenofibrate use and the RNFL thickness of the right eye in patients with type 2 diabetes

Risk factors	B	S.E.	Sig.	95%CI for OR	
				upper	lower
Fenofibrate	3.225	1.605	*0.042	0.027	6.424
BMI	−3.475	1.611	*0.034	−6.678	−0.271
ACR	−0.650	0.281	*0.024	−1.210	−0.090
TC	−5.584	−0.269	0.538	−23.58	12.41
TG	−0.779	2.745	0.777	−6.248	4.689
HDL	9.329	13.320	0.486	17.211	35.869
LDL	6.834	10.310	0.510	13.710	27.378
HbA1c	1.382	1.703	0.420	−2.012	4.776
Diabetic duration	−0.337	0.484	0.488	−1.300	0.627
AL	−1.318	0.720	0.061	0.809	15.013
IOP	−0.547	0.550	0.310	0.126	1.573

*BMI* body mass index, *DR* diabetic retinopathy, *HbA1c* glycated hemoglobin, *HDL* high-density lipoprotein, *LDL* low-density lipoprotein, *TC* Total cholesterol, *TG* Triglyceride, *ACR* urinary albumin-to-creatinine ratio, *RNFL* retinal nerve fiber layer. *S.E*.standard error. *Sig*. significant. *CI* Confidence Interval, *OR* Odds Ratios. The multiple liner regression models were adjusted by age, gender, diabetic duration. * *p* < 0.05. A *p* value < 0.05 was defined as statistically significant

## Discussion

The present study investigated the thickness of RNFL at early stage of DR in diabetic patients whom were treated with or without fenofibrate. The results demonstrated that patients who did not use fenofibrate had a thinner superior quadrant RNFL thickness than fenofibrate user, when the results were adjusted for age, gender, AL, IOP, DR status, ACR and medication for DM treatment. Regarding the potential risk factors for RNFL loss, we found that the effect of fenofibrate on RNFL loss was not likely related to its lipid-lowering effect, but might be associated with its ability to regulate vascular endothelial function. This notion, however, needs to be examined by further studies.

DR is considered to be manifested by neurodegenerative changes at early stage before vascular abnormality occurs. Accumulating clinical and experimental studies have shown that neuronal abnormalities and apoptosis of different types of neuronal cells appeared in all retinal layers at early stages of DR [22, 23], and RNFL thinning was considered to be an important changes in diabetic retinal neurodegeneration and related to the severity of DR [24] . Early intervention was the only effective way to delay irreversible vision loss [25]. As one type of the most widely used lipid-lowering agents, fibrates have been reported to prevent DR progression and to reduce the need for laser treatment [4, 6] [26–29]. However, the correlation between fenofibrate use and retinal neurodegeneration in diabetic patients has not been reported yet. The present study therefore investigated the relationship between use of fenofibrate and the reduction in risk for RNFL loss at early stage of type 2 DM. We found that oral administration of fenofibrate could prevent RNFL loss in the superior quadrant of the right eye in diabetic patients without DR or with NPDR. Patients who never used fibrates had reduced RNFL thickness than those who were administrated with fenofibrate. Choi et al. [30] reported that RNFL defects in type 2 DM occurred more frequently on the superior side of retina (75.6% and 71.0% in right and left eyes, respectively). Lopes et al. [31] also reported that RNFL thickness of the superior segment became significantly thinner in patients with type 1 diabetes without DR than in normal population. Therefore, the superior quadrant of RNFL might represent the earliest and most significantly affected region in the retina; decrease in thickness of this area could be detected with SD-OCT and be used as an important indicator for assessing the effects of fenofibrate.

Fibrates are orally administered fibric acid derivatives that are conventionally used alone or as an adjunct to statins in treating dyslipidemia [32]. To assess whether the effect of fenofibrate on RNFL loss was related to its lipid-lowering effect, we performed a linear regressions analysis for the association between RNFL thickness and blood lipids levels. However, no significant difference was found between the level of any type of blood lipid and the superior quadrant RNFL thickness of the right eye. The results suggested that lowering the serum lipids levels might not represent the mechanism accounting for fenofibrate’s effect on RNFL loss. On the contrary, BMI was found negatively correlated with the superior RNFL thickness of the right eye, therefore, we suggested that restrict control of body weight might be another protective factor for delaying diabetic retinal neurodegeneration in DM patients.

Concerning other factors that might have an effect on RNFL thickness and were not well-matched between two groups, we found that ACR was negatively related to the superior RNFL thickness. ACR was regarded as an indirect indicator of endothelial function [33–35]. Some researchers considered that diabetic retinal neurodegeneration was partially associated with endothelial dysfunction by decreasing blood supply to the optic nerve head [30] [36]. A vascular insufficient optic nerve head might result in RNFL thinning in the optic disc on the superior side because of the gravitational influence. Therefore, vascular endothelial dysfunction represented the main pathophysiology of diabetes and was closely related to the severity of DR and retinal neurodegeneration [37]. We presumed that the effect of fenofibrate on RNFL loss might be partially related to endothelial dysfunction in patients with type 2 DM. However, the specific mechanism still need further study with animal experiments.

Several studies have shown that fibrates could affect signaling pathways involved in inflammation [38], angiogenesis [39] and cell survival [40], such as AMPK pathway [41] that plays important roles in diabetic vascular dysfunction and neurodegeneration. Therefore, we hypothesized that oral administration of fenofibrate might prevent RNFL loss through adjusting the endothelial dysfunction in retina; this could improve blood flow in vessels and reduce vascular leakage [42], and therefore increase the blood supply of the optic disc to avoid cell death. In addition, we also hypothesized that fibrates might delay neuron death and glial cell reactivation through its anti-inflammation and anti-apoptosis properties [43], which needs to be proved by further clinical and experimental studies. However, due to the limitations of observational study, we couldn’t assess the direct relationship between fenofibrate administration and ACR fluctuation, which might be completed by a well-matched prospective cohort study in future.

There were several limitations in the present study. Firstly, this was a case-control study, which mainly explored the association between use of fibrates and diabetic RNFL loss but did not take into account the dose of fibrates. Secondly, we didn’t perform subgroup analyses to compare the patients who used fibrates regularly and irregularly. Thirdly, we couldn’t collect the multifocal electroretinogram data of all the subjects, which was considered another effective method to assess neurodegeneration in retina [44], because patients at early stage of DR were not requested to do this examination.

## Conclusions

Oral administration of fenofibrate was suggestively associated with thicker RNFL in superior quadrant of the right eye of patents with early DR.

## Abbreviations

AL: Axial length
BMI: body mass index
DM: Diabetic mellitus
DR: diabetic retinopathy
DR: Diabetic retinopathy
HbA1c: Glycated hemoglobin
HDL: High-density lipoprotein
IOP: Intraocular pressure
LDL: Low-density lipoprotein
RNFL: Retinal nerve fiber layer
